# Successful Treatment of Secondary L5 Osteoporotic Vertebral Fracture Post-balloon Kyphoplasty With Revision Balloon Kyphoplasty

**DOI:** 10.7759/cureus.68995

**Published:** 2024-09-09

**Authors:** Keita Kuraishi, Yoshinori Maki, Yoshihiko Ioroi, Tamaki Kobayashi

**Affiliations:** 1 Spinal Surgery, Ohmi Sebone Clinic, Ohmihachiman, JPN; 2 Neurosurgery, Ayabe Renaiss Hospital, Ayabe, JPN; 3 Neurosurgery, Hikone Chuo Hospital, Hikone, JPN; 4 Spinal Surgery, Kyoto Katsura Hospital, Kyoto, JPN

**Keywords:** balloon kyphoplasty, lumbar, osteoporotic vertebral fracture, pincer type fracture, posterior fixation surgery

## Abstract

Balloon kyphoplasty (BKP) is a standardized, minimally invasive procedure for treating osteoporotic vertebral fractures. However, secondary osteoporotic vertebral fractures following BKP are uncommon in clinical practice, and there is limited published experience with revision BKP for this condition. An 82-year-old man presented to our clinic with back pain after a fall. Computed tomography and magnetic resonance imaging revealed an osteoporotic L5 vertebral fracture. As conservative treatment was unsuccessful, he underwent BKP for the L5 fracture, which alleviated his pain. Twenty days post-operatively, the patient developed renewed back pain. Subsequent imaging demonstrated a secondary osteoporotic fracture within the previously treated L5 vertebra, involving both the upper and lower endplates. While posterior fixation was considered, the patient declined surgery. Therefore, we performed a revision BKP to address the secondary fracture. The postoperative course was uneventful, and 10 months later, computed tomography images showed evidence of bone healing and remodeling in the L5 vertebral body. Our case suggests that revision BKP may be an effective treatment option for secondary osteoporotic vertebral fractures. However, careful patient selection is crucial to ensure the safety and efficacy of this procedure.

## Introduction

Osteoporotic vertebral fractures are a prevalent and growing public health issue in aging populations [1]. While conservative treatments like external fixation, medications, and rehabilitation can be effective for patients with mild symptoms, surgical intervention is often necessary for those with severe pain or neurological deficits [2]. Given the elderly patient population typically affected by these fractures, minimally invasive surgical options are highly desirable.

Balloon kyphoplasty (BKP) is considered a minimally invasive procedure for treating osteoporotic vertebral fractures. It involves percutaneous insertion of balloons through the pedicles into the fractured vertebral body. After balloon expansion, cement is injected to stabilize the collapsed vertebra. Despite potential risks such as cement leakage, delirium, infection, pulmonary embolization, and the need for revision surgery, BKP is widely performed due to its effectiveness in pain relief and improved quality of life [3-5]. When a secondary vertebral fracture occurs after BKP, traditional fixation surgery is an option. However, successful revision BKP for such fractures is rarely reported [6].

This report presents a rare case of a secondary osteoporotic L5 vertebral fracture following BKP that was successfully treated with a revision BKP.

## Case presentation

**Figure 1:**
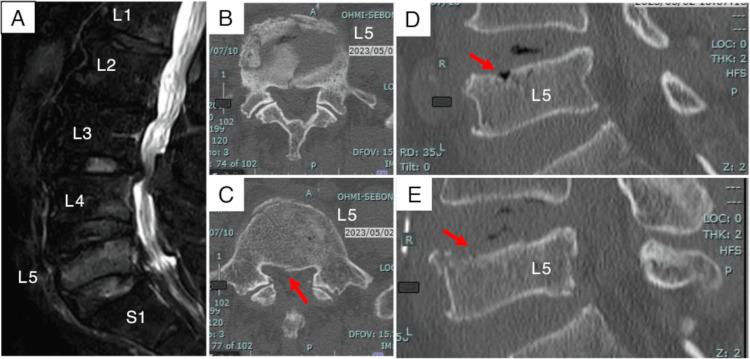
Initial radiological findings (A) A sagittal short tau inversion recovery image shows a high-intensity lesion in the L5 vertebral body. (B, C) Axial computed tomography images demonstrate an upper vertebral endplate defect and a posterior wall fracture (red arrow). (D, E) Sagittal computed tomography images confirm the upper vertebral endplate defect.

An 82-year-old fisherman with chronic back and left knee pain underwent treatment at our outpatient clinic. Three months into his medication regimen, he presented with acute, severe back pain after a fall and impact to his back. Radiographic imaging revealed an osteoporotic L5 vertebral fracture with an associated upper vertebral endplate defect and posterior wall fracture (Figure 1).

Despite pain management with a Damen corset and medication, surgical intervention was considered necessary due to persistent pain. The risk of additional surgery for endplate defects prompted us to explore less invasive options [3]. Given the patient’s refusal of fixation surgery, BKP was planned.

**Figure 2:**
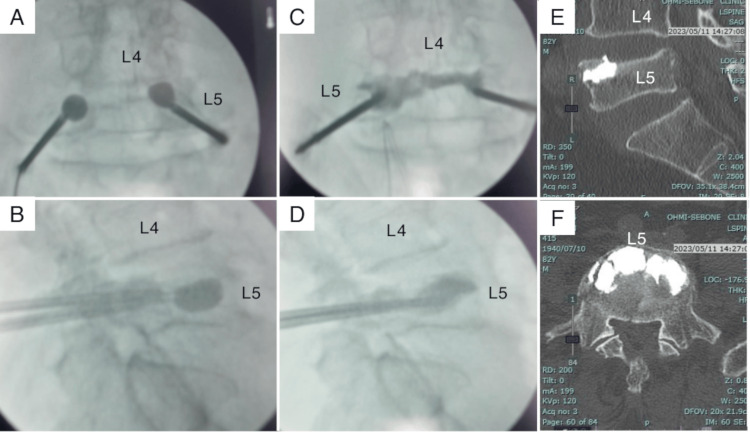
Intraoperative and postoperative images of the initial balloon kyphoplasty (A, B) Balloons were transpedicularly inserted near the fracture. (C, D) A total of 7.3 mL of cement was injected into the vertebral body. (E, F) Postoperative computed tomography images (anteroposterior and lateral projections) confirm cement placement near the fracture.

Twenty days post-fall, BKP was performed. To prevent cement leakage, due to the existing posterior wall fracture, cement was injected anteriorly. Two balloons were inserted bilaterally, 5 cm lateral to midline, and 7.5 mL of cement was injected targeting the anterior fracture line (Figure 2).

**Figure 3:**
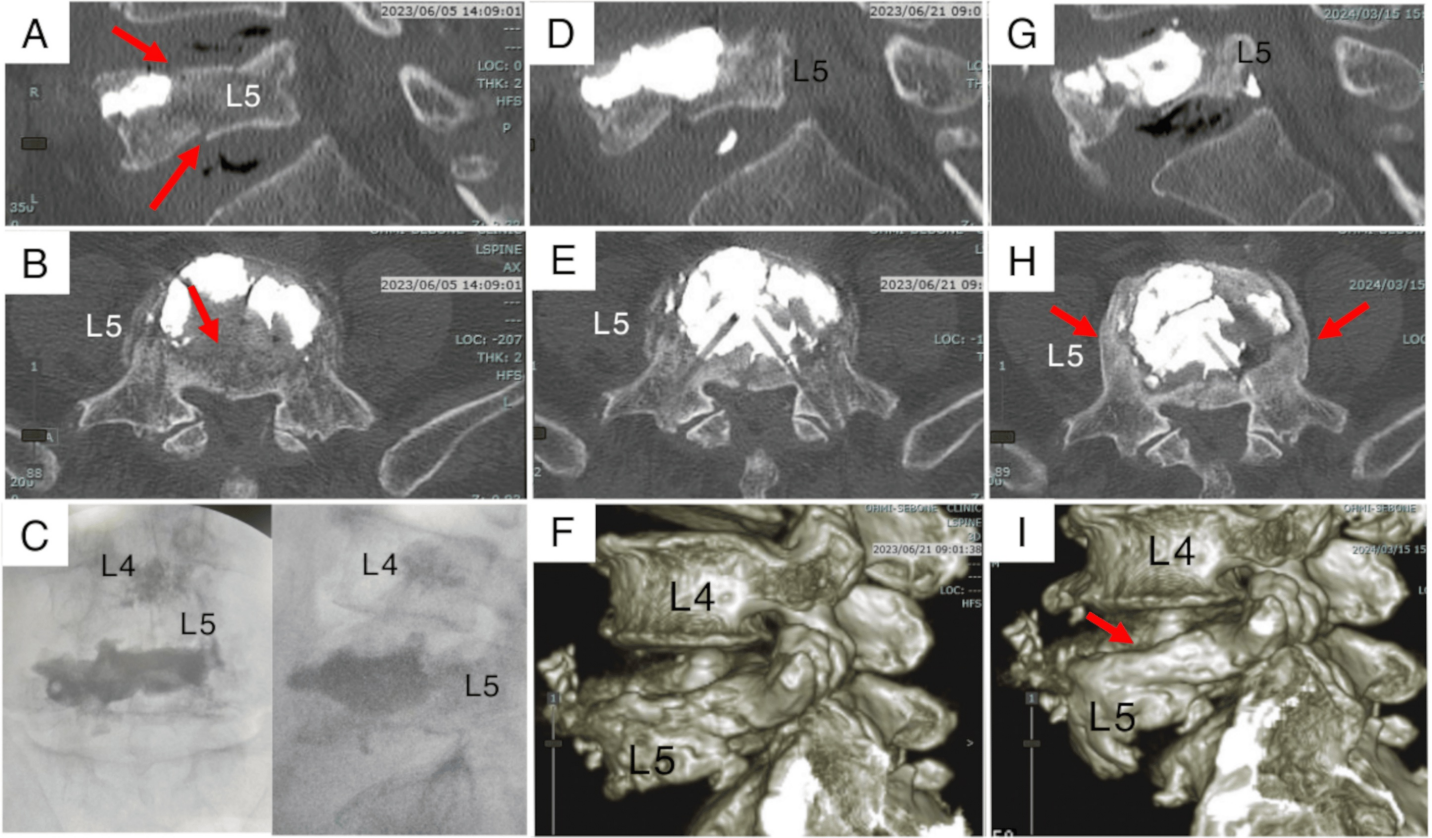
Preoperative and postoperative images of the revision balloon kyphoplasty (A, B) A new fracture (red arrows) is evident dorsal to the cement from the initial balloon kyphoplasty. (C) An additional 9 mL of cement was injected during the revision balloon kyphoplasty. (D, E) The additional cement covers the secondary fracture. (G, H) Ten months post-revision, osteogenesis and remodeling (red arrows) are observed in the lateral wall of the L5 vertebral body. (F, I) Three-dimensional reconstructed computed tomography images demonstrate bony fusion.

Initial pain relief was followed by recurrent back pain 20 days post-BKP, without a new fall. Computed tomography demonstrated a secondary osteoporotic L5 vertebral fracture located dorsally to the injected cement (Figures 3A, 3B), classified as a pincer-type fracture according to the DGOU (German Society for Orthopaedics and Trauma Surgery) osteoporotic fracture classification system [1,7]. Despite the patient’s aversion to surgery, the severity of the new fracture warranted intervention.

Following informed consent, a revision BKP was performed one month later. Balloons were inserted more laterally (8 cm) and expanded both ventrally and dorsally to address the new fracture, with a total of 9 mL of cement injected (Figures 3C-3F). Postoperative pain resolved, and the patient was discharged independently the following day. Ten-month follow-up revealed bone healing and remodeling in the lateral L5 vertebral body without complications (Figures 3G-3I).

## Discussion

This report presents a case of secondary osteoporotic L5 vertebral fracture following BKP successfully treated with revision BKP. Given the patient’s refusal of fixation surgery, BKP was the only treatment option throughout the clinical course. The revision BKP was effective in resolving back pain related to the secondary fracture. As similar reports seem not to be often described [6], this is a rare successful case of a revision BKP resolving a secondary osteoporotic vertebral fracture.

The initial L5 fracture involved the upper endplate deficit and posterior wall. While severe posterior wall fractures are typically contraindicated for BKP due to the risk of cement leakage [3,8], the relatively mild deformity in our case allowed for anterior cement placement. Although initially effective, a secondary fracture subsequently occurred.

Takahashi et al. identified the upper endplate defect as a risk factor for revision BKP [3], a condition present in our patient. Moreover, the dorsal location of the secondary fracture relative to the injected cement suggests that a rigidity gap between the cement and bone marrow may have contributed to mechanical stress and fracture formation.

The secondary osteoporotic L5 vertebral fracture was morphologically classified as a pincer-type fracture according to the DGOU osteoporotic fracture classification system [1,7]. Due to the patient’s severe pain-induced limitations in mobility, surgical intervention was deemed the most appropriate course of action. However, the patient refused fixation surgery for the second time.

Not performing surgery for the secondary fracture and opting for conservative treatment alone could have delayed or worsened the patient’s recovery of daily activities. After explaining the potential need for fixation surgery following revision BKP due to possible vertebral deformity progression, we obtained informed consent from the patient and their family. In the revision BKP, the cement was injected both ventrally and dorsally to encompass the longitudinal fracture. A total of 9 mL of cement was successfully injected into the vertebral body, leading to osteogenesis and remodeling of the L5 vertebral body.

Yonezawa et al. reported on three cases of thoracic and lumbar osteoporotic vertebral fractures that necessitated revision BKP for secondary osteoporotic vertebral fractures. Their revision BKP cases involved one patient with multiple fractures at baseline, another with fractures at adjacent levels, and a third with a secondary fracture at the same level. Notably, the last case underwent revision BKP guided by a cannulated screw [6]. Therefore, our case is unique in that a revision BKP, without any additional procedure (i.e., a cannulated screw), effectively resolved a secondary osteoporotic vertebral fracture at the same level that had been treated with the initial BKP.

This study is limited by its single-case design and a relatively short follow-up period of 10 months. Longer-term outcomes are necessary to assess the durability and efficacy of revision BKP for secondary osteoporotic vertebral fractures. Further studies with a larger cohort are required to establish standard indications for this procedure. A standardized protocol for patient selection and surgical technique should be developed based on accumulating clinical cases. Given the potential for complications, having a contingency plan in place, such as the ability to perform fixation surgery, is crucial for managing unexpected events during and after revision BKP.

## Conclusions

We reported a case of a secondary osteoporotic L5 vertebral fracture successfully managed with a revision BKP. As observed in our case, this surgical strategy may be effective in resolving the secondary osteoporotic L5 vertebral fracture. However, this is a single case with limited follow-up, and revision BKP is not yet a gold standard treatment for secondary osteoporotic L5 vertebral fractures. It may offer an alternative to fixation surgery in carefully selected patients, but strict patient selection and careful surgical planning are essential.
